# Loneliness and social isolation: exploring the experiences of older women during the pandemic in terms of social connection, feeling of loneliness, and the impact on mental health and wellbeing

**DOI:** 10.3389/fgwh.2024.1410058

**Published:** 2024-06-28

**Authors:** Nasrullah Bhat, Fayaz Ahmad Paul, Aamir Gul, Zahoor Ahmad Ganie

**Affiliations:** ^1^ Department of Social Work, University of Kashmir, Srinagar, India; ^2^ Department of Psychiatric Social Work, LGB Regional Institute of Mental Health, Tezpur, India; ^3^ Institute of Kashmir Studies, University of Kashmir, Srinagar, India; ^4^ Department of Social Work, Govt. Degree College for Women, Srinagar, India

**Keywords:** loneliness, social isolation, older women, COVID-19 pandemic, mental health, social connection

## Abstract

**Objectives:**

To investigate the unique challenges faced by older women during the COVID-19 pandemic regarding social connection, feelings of loneliness, and their subsequent impact on mental health and well-being.

**Method:**

A qualitative research methodology is used to examine how older women experienced loneliness, social relationships, and mental health consequences during the COVID-19 pandemic.

**Results:**

The results are discussed in terms of two main themes each with their sub-themes; 1. Social Connection amidst Physical Distancing Measures, 2. Impact on Mental Health and Well-being.

**Conclusion:**

The study highlights the significant impact of loneliness and social isolation on the mental health of older women during the COVID-19 pandemic, emphasizing the need for targeted interventions and support systems.

## Introduction

Social relationships, mental health, and general well-being have all been significantly impacted by the COVID-19 pandemic, with older people being more susceptible to its negative consequences (1, 2). Given the intersections of age, gender, and societal roles that influence older people's experiences in times of crisis, women represent a demographic segment among them that faces particular obstacles (3). Due to the pandemic, loneliness and social isolation among older women have become more prominent, emphasising the need for more study to fully comprehend the intricacies of these women's experiences negotiating relationships with others, feelings of loneliness, and mental health consequences (4). It has long been known that loneliness and social isolation, especially in older people, are important risk factors for poor mental health outcomes, such as depression, anxiety, and cognitive decline (5). Social networks have been disrupted and possibilities for face-to-face encounters have been limited as a result of the pandemic's physical distancing measures and lockdowns (6). These tactics have contributed to feelings of loneliness and isolation. There is still a dearth of studies explicitly looking at older women's experiences with loneliness, social isolation, and mental health outcomes during the COVID-19 pandemic, despite the increasing awareness of the pandemic's effects on older adults' mental health (7). Studies that have already been done frequently don't take a gender-sensitive stance, ignoring the particular social roles, caregiving obligations, and structural injustices that older women face (8, 9).

More so than other factors, gender has a major influence on how older persons perceive social relationships and loneliness. Studies reveal that gendered norms, social positions, and personal encounters might impact an individual's perspective and encounters with loneliness, in addition to the calibre and character of their social ties (10–12). Social roles and expectations are associated with one gendered aspect of loneliness. In contrast to males, women have historically been socialised to value relational and caring responsibilities, which has resulted in greater social networks and support systems (13). However, these social roles and connections can be upset as people age by life transitions including widowhood, retirement, and health problems. This can exacerbate feelings of loneliness and isolation, especially for older women (14). Furthermore, the expectations that society has regarding gender roles may have an impact on older women's desire to ask for assistance or reveal feelings of loneliness, which might result in them underreporting or hiding their emotional difficulties (14). Furthermore, gender differences may exist in the type and quality of social connections. According to research, older women are more prone to depend on intimate friends and family interactions for companionship and social support (15). The significance of social relationships for the mental health and overall well-being of older women has also been highlighted by the COVID-19. Studies indicate that in times of crisis, older women could mostly depend on social networks for emotional support, company, and coping mechanisms (16). However, physical distancing tactics and lockdowns have broken these relationships with others, making older women feel more isolated and distressed psychologically (17). Additionally, older women's experiences of loneliness and isolation have been exacerbated by the loss of social support networks brought on by sickness, bereavement, or migration during the pandemic, aggravating pre-existing mental health issues (18). The COVID-19 epidemic has brought attention to the gendered aspects of social connections and loneliness among older women, highlighting the need for gender-sensitive strategies to reduce social isolation and foster resilience in mental health. Gendered norms around socialisation and communication may also have an impact on older women's perceptions of loneliness during the epidemic. According to research, males frequently rely on more utilitarian, task-oriented connections, whereas women prioritise and value personal, emotionally expressive relationships (19). Because of this, physical separation measures and lockdowns may cause older women more grief when their possibilities for in-person connections and emotional support are limited. Furthermore, compared to males, older women may feel more comfortable sharing their experiences of loneliness and reaching out for social assistance because of gendered standards around emotional expression (20). Still, some older women may be discouraged from openly addressing their challenges or seeking professional support due to the stigma associated with mental health concerns and loneliness (21). These findings have led to an increased awareness that older women's experiences throughout the COVID-19 pandemic should be more extensively studied. It is necessary to understand how age, sex and gender intersect with social determinants of health if we are going to create interventions and support systems tailored for this population. Consequently, what this research hopes to achieve is investigating what happened during those times among older females regarding such aspects as mental wellness or even better health; taking into account such topics like socialization, loneliness etc.? We can therefore gain a deeper understanding of the lives they live through qualitative methods which will enable us also come up with ways on how best we can enhance their resilience in times of crisis while living healthier lives.

However, this study has been carried out in Indian part of Jammu and Kashmir. The region of Jammu and Kashmir in India has different cultural and social norms than the rest of India. This history, socio-political contexts, and cultural traditions have an impact on how its people live. For example, social isolation and loneliness might be perceived differently in a society like Kashmir's having tight-knit family structures and community support. However, extended conflict and instability in the area can also result in more stress and mental health problems. The level of egalitarianism in J&K may differ from place to place as well. Nevertheless, some parts of the Kashmiri society are progressive such as support for women's roles while others remain conservative as always. This dissonance is what makes it difficult to study older women's experiences since they may be under supportive family relations whilst confined by societal expectations. With its unique cultural and socio-political context, the experiences and accounts of participants of this study might be influenced. For example, the emphasis on family and community may mean that older women in J&K cope with loneliness differently from their counterparts in other parts of India or even the whole world. Moreover, ongoing conflicts in this area could heighten feelings of being alone and worried thus affecting mental well-being different from anywhere else.

## Materials and methods

With a focus on capturing the complexity and depth of participant experiences, a qualitative research methodology is used to examine how older women experienced loneliness, social relationships, and mental health consequences during the COVID-19 pandemic. Researchers examined subjective meanings, viewpoints, and circumstances through participants lived experiences by using qualitative approaches, which are ideal for analysing complex social phenomena (22). Researchers were able to gain a clearer understanding of the intricate interactions between gender dynamics, societal determinants, and older women's mental health by using an interpretive perspective. The thematic analysis used for the above study is primarily inductive thematic analysis.

### Participant recruitment

In qualitative research, it is common to conduct interviews with a relatively small number of participants, typically ranging from 10 to 30 individuals (23). The study has been carried out in Indian Administered Kashmir. The present study recruited 15 participants purposively. In recruiting participants for the study exploring the experiences of older women during the COVID-19 pandemic, several recruitment strategies were employed to ensure diverse representation and capture a range of perspectives. These strategies include:

Community Organizations: Collaborating with community organizations that serve older adults, such as senior centers, retirement communities, or support groups, can facilitate access to potential participants.

Healthcare Settings: Partnering with healthcare providers or clinics that cater to older adults can help identify individuals who may be interested in participating in the study.

Online Platforms: Utilizing online platforms and social media channels to disseminate recruitment notices or advertisements can reach a broader audience of older women who may be interested in sharing their experiences.

### Data collection

Prior to data collection, participants were provided with detailed information about the study, including its purpose, procedures, potential risks, and benefits. Participants were given the opportunity to ask questions and provide informed consent before participating in the study. The study utilized rigorous data collection procedures, such as semi-structured interviews conducted by trained researchers. Probing questions were also used to elicit detailed responses and ensure thorough exploration of participants' experiences. The researchers used purposive sampling to recruit participants for the present study. This focused the study on individuals with different backgrounds and outlooks. At every stage of data gathering, researchers of this study analyzed information by coding interview transcripts and sorting them into groups based on similar ideas or recurring themes. Researchers did this over and over again until it seemed like new material no longer offered fresh knowledge about what they were investigating. When interviews started sounding repetitive because interviewees began echoing each other's words or descriptions too closely, researchers knew they had reached thematic saturation; when nothing new came up in later conversations besides what had already been mentioned earlier, researchers understood that additional talks would not produce any more categories than those already identified or supply redundant details to what was already known so far.

### Data analysis

The qualitative data analysis for the study on the experiences of older women during the COVID-19 pandemic utilized an inductive thematic analysis approach, drawing on principles of Interpretative Phenomenological Analysis (IPA) to explore participants' narratives in-depth and generate themes (24). It involves:

(a) Familiarization with the Data: The analysis process began with immersion in the interview data to gain familiarity with the content and context of participants' narratives. This involved repeated readings of the transcripts and detailed note-taking to capture key ideas, phrases, and expressions related to loneliness, social connections, and mental health experiences during the pandemic.
(b) Initial Coding: Initial coding involved the systematic identification and labelling of meaningful units of text relevant to the research questions. Codes were generated inductively from the data, focusing on specific aspects of participants' experiences, emotions, and coping strategies related to loneliness, social isolation, and mental well-being. This process allowed for the exploration of diverse perspectives and nuances within the dataset.
(c) Generating Categories: Following initial coding, similar codes were grouped together to form preliminary categories or clusters of meaning. These categories emerged organically from the data and reflected common themes and patterns observed across multiple interviews. Categories were flexible and iterative, with adjustments made as new data were analysed and additional insights emerged.
(d) Theme Development: Through a process of iterative refinement and comparison, categories were further distilled into overarching themes that captured the essence of participants' experiences and perspectives. Themes were characterized by coherence, relevance, and resonance with the research objectives, reflecting shared patterns of meaning and significance across the dataset. Themes were supported by rich illustrative quotes from participants' narratives, enhancing the credibility and depth of the analysis.
(e) Interpretation and Reflexivity: Throughout the analysis process, attention was paid to the researchers' interpretations and reflexivity, acknowledging the influence of their own perspectives, biases, and preconceptions on the analysis. Reflexive journaling and peer debriefing were used to critically examine and challenge interpretations, ensuring that the themes accurately represented participants' voices and experiences.
(f) Finalizing the Analysis: The final stage of analysis involved refining and consolidating the identified themes into a coherent narrative that provided insight into the complexities of older women's experiences during the pandemic. The themes were contextualized within the broader literature on loneliness, social connections, and mental health, highlighting their theoretical and practical implications for understanding and addressing the psychosocial impacts of the pandemic on older women.

**Table 1:** Participant demographic information.

Participant	Age	Gender	Marital status	Living arrangement	Educational level
P1	65	Female	Married	With spouse	High School
P2	69	Female	Married	With spouse	Bachelors
P3	75	Female	Married	With spouse	Bachelors
P5	68	Female	Widow	With son	Masters
P6	75	Female	Widow	With daughter	Bachelors
P7	73	Female	Widow	With brother	High School
P8	72	Female	Married	With spouse	High School
P09	65	Female	Married	With spouse	Bachelors
P10	64	Female	Widow	With son	Bachelors
P11	66	Female	Married	With spouse	Masters
P12	68	Female	Married	With spouse	Masters
P13	70	Female	Married	With spouse	Bachelors
P14	72	Female	Married	With spouse	Bachelors
P15	65	Female	Divorcee	With daughter	High School
P16	66	Female	Married	With spouse	Bachelors

### Ethical consideration

The study adhered all the ethical considerations necessary to carry out the study. Respecting participants' autonomy, choices, and cultural beliefs throughout the research process, including their right to withdraw from the study at any time without repercussion was highlighted in the written Informed Consent. In adherence to principles of confidentiality, participant names were anonymized throughout the qualitative research process. As seen in the Table 1, codes were assigned to each participant to protect their identity and ensure their privacy. Researchers implemented measures to minimize potential harm or distress to participants, such as providing access to mental health resources and support services if needed, and ensured that research findings are communicated in a responsible and sensitive manner.

## Results

The analysis of the data resulted into two main themes each with their sub-themes

1. Social Connection amidst Physical Distancing Measures
  1.1 Embracing Technology for Virtual Interactions
  1.2 Rediscovering the Value of Intimate Relationships
  1.3 Navigating Changes in Social Routines
2. Impact on Mental Health and Well-being
  2.1 Struggles with Anxiety and Depression
  2.2 Coping through Creative Expression and Self-Care
  2.3 Finding Strength in Resilience and Social Support

### Social connection amidst physical distancing measures

Amid the COVID-19 pandemic and other global crises, social connection is both essential and complicated. The themes we are seeking within the theme “Social Connection amidst Physical Distancing Measures” illustrate the fine line between the need for human connection and the life-saving importance of following physical distancing rules to keep from spreading the virus. This theme illuminates how people—including and especially older women—are managing to keep their social ties close, maintain relationships, and keep feelings of isolation at bay as they face extraordinary challenges.

#### Embracing technology for virtual interactions

“Embracing Technology for Virtual Interactions” appears as a crucial sub-theme in the age of physical separation, emphasising the transformational power of digital communication in mending social barriers and promoting belongingness among older women. Steep learning curves often accompany the virtual world. The corona pandemic forced technology on a 75-year-old grandmother;

I'm committed to maintain contact with my grandkids because they mean the world to me. I gained proficiency in video calls. Social media welcomed me with open arms, much like old friends do. I had little experience with technology at first, but I quickly became adept at using the internet. Ours became into rituals: online cookery lessons and virtual Storytime. However, even though I was hundreds of miles away, I was present to celebrate their victories and threshold crossings.

The narrative captures the experience of several older women who, in the midst of the epidemic, relied on technology to stay in touch with one another. According to research, older adults—including older women—have embraced social media and video chats as digital communication tools to remain in touch with friends and family (25). Similarly another retired government women employee participant mentioned;

These digital interactions have served as a source of solace and strength during challenging circumstances, underscoring the notion that amidst despair, a glimmer of hope can be discovered emanating from the luminosity of a computer display.

The narrative emphasises the value of intergenerational support in helping older individuals embrace technology. Grandchildren frequently play a significant role in offering advice and support while using digital gadgets. The story also emphasises the positive emotional effects of virtual relationships, as older persons experience happiness and connection when interacting with loved ones via technology (26). Another participant, 68, narrated;

Through technology, I learned that love can transcend boundaries and generations, bringing individuals even closer to one another. These virtual connections gave me support and fortitude throughout trying times, proving that even at the darkest moments, there can be hope found in the brightness of a computer screen.

The narrative highlights the difficulties and successes faced by older women navigating the internet world to find social connections. Studies indicate that technological access and use may be impeded for older persons, particularly those who are single or socially isolated, due to factors including low digital literacy and worries about security and privacy (27). To fight loneliness and preserve social ties throughout the epidemic, the narrative emphasises how resilient and adaptive older women were in embracing new technology.

#### Rediscovering the value of intimate relationship

The sub-theme “Rediscovering the Value of Intimate Relationships” explores the emotional journey that older women go through in the midst of the COVID-19 epidemic, exploring the intricacies of love, grief, and connection. The topic in question looks at how, in the face of social isolation, bereavement, and resilience, older women redefine and reassess the value of close connections, whether they be with spouses, family, or friends. For example one of the participant who is 69 years old mentioned;

I have seen the strength and resilience of family ties which anchor us through the storm. One time during the pandemic, loved ones hugged me, and it is those close contacts that really make us feel alive. We are linked by our unshakable bonds of love and brotherhood and came through the pandemic’s trials together, not worse but stronger.

The narrative highlights the resilience and ingenious coping mechanisms of older women in the face of loss, emphasising the need of fostering close relationships and finding comfort in familial ties in the midst of grief and loneliness. Studies indicate that preserving relationships with loved ones might lessen loneliness and enhance mental health among senior citizens (15). Another participant who is 65 years old divorcee narrated;

I realized that real intimacy comes from one's feeling of oneself and needs real healing and growth. Being able to truly accept myself as I am allows me to form more substantial, respectful relationships with others. I am not really a human being, but a living organism; this is also my idea.

This narrative illustrates the self-care and self-discovery journeys older women undertook in the face of the pandemic's obstacles. According to secondary research, the pandemic presented particular emotional and psychological issues for older adults, such as feelings of loneliness and existential doubt, including divorcees and single people (28).

#### Navigating changes in social reform

The sub-theme “Navigating Changes in Social Reform” explores the challenges experienced by older women in the wake of the COVID-19 epidemic as they adjust to changing roles, expectations, and social norms. To shed insight into older women's resilience, ingenuity, and agency in facing and influencing social reforms during times of crisis, this subject examines how they negotiate changes in societal structures, community dynamics, and support networks. As the 68 years old widow manifested;

Caring for the older is not as straightforward as you might think. Right now, the outbreak makes it abundantly clear how tense social structures are, and also how the injustices inside them still exist. I felt isolated and overlooked when places of senior citizens support service were shut down for business: this underscores a critical need for reform from within. I banded together with other advocates for things like equality amongst old-age groups and put my efforts into generating support for social programs so that the voice of the older could be heard. By putting out letters, stirring up online demonstrations, and going door to door to contact older ladies, we made people aware of their problems and picked up the concept of making neighbourhoods friendlier toward old folks. Though social transformation faces many difficulties, I am confident that our joint endeavours will succeed in making society more inclusive.

The narrative demonstrates the proactive stance elder women took in supporting social transformation in reaction to the difficulties the epidemic presented. The main character exhibits agency and fortitude in the face of societal shifts, using her connections and abilities to affect significant change in her neighbourhood. This account is consistent with secondary evidence showing that older adults—particularly older women—have actively supported social reform and spearheaded community resilience initiatives throughout the epidemic (29). The narrative emphasises the agency and determination of older women to take on societal issues and bring about positive changes in their local communities, underscoring the significance of intergenerational cooperation and coordinated effort in advancing social transformation. Another participant who is 65 years old working as a principal in the local school mentioned;

How the epidemic made me think about in way that societal norms and standards influence. Without a doubt, my sense of community and belonging was thoroughly examined by a rapid change to remote working with almost no connection; I felt lost and uneasy– However, in midst of chaos, my colleagues' perseverance and our united voice gave me some comfort. We came together against ageism and for the promotion of increased accessibility and inclusiveness as well as respect for all people in our communities.

The emotional and psychological effects of societal shifts on older women's sense of identity and belonging during the epidemic are depicted in this narrative. Studies indicate that in the face of changing societal norms and institutions, older adults—especially those who are single or facing social isolation—may have increased emotions of isolation, unease, and alienation (30). However studies also demonstrate that to overcome these obstacles and preserve their wellbeing, older persons frequently rely on their resiliency and coping mechanisms, such as reaching out to others for support and taking part in worthwhile activities (31). The narrative emphasises how crucial it is to foster embracing, caring communities that understand and cater to the many needs of senior women, especially in times of distress.

### Impact on mental health and wellbeing

The theme of “Impact on Mental Health and Well-Being” explores the deep emotional impact that the COVID-19 epidemic took on people, especially older women. Amid unparalleled uncertainty and upheaval, this theme delves into the complex issues, coping techniques, and resilience tactics older women use to negotiate the interconnected aspects of mental health, loneliness, and social isolation. The topic illuminates the intricate interplay between social and psychological elements that shape older women's mental health and well-being during times of crisis via accounts of personal hardships, coping mechanisms, and paths to resilience.

#### Struggles with anxiety and depression

The sub-theme “Struggles with Anxiety and Depression” explores the severe emotional difficulties that older women encountered during the COVID-19 epidemic. Examining the variables that contribute to their mental health issues, the coping strategies they use to overcome hardship, and the routes to recovery and resilience, this subject delves into the complex experiences of anxiety and depression among older women. The 66 years old women narrated;

I had never been flooded with so much fear and so much sorrow as during the COVID-19 epidemic. I was helpless and debilitated by news of the terrifying reality, and the virus was hanging over me like the sword of Damocles.

This account is consistent with secondary research showing that older persons are more likely to experience anxiety and sadness during the pandemic, especially if they are retired and living alone (32). The aforementioned example illustrates how social isolation, routine disruption, and fear of disease all raise psychological distress levels in older persons at times of crisis. Another 68 years old widow mentioned;

I seemed to be stuck in a state of anxiety and sadness thanks to the pandemic. In the aftermath of my partner’s sudden death, I was just as afraid of loneliness as I was of death.

The narrative emphasises the significant impact of worry, uncertainty, and social isolation on older women's' mental health during the pandemic. According to research, older persons who suffer from losses like the death of a spouse are more likely to have anxiety and depression symptoms, especially when they are socially isolated and have little social support (33). A 75 year old retiree women narrated;

Because of the pandemic, my mental health deteriorated, and as a result, I struggled with anxiety and sadness. My community's once-bustling streets had become disturbingly silent, reflecting the emptiness inside of me. Thoughts of what was to become of them devoured my being, which led to an underlying feeling of despair. This was the situation

These individual accounts illustrate the significant challenges that older women are facing as they deal with depression and anxiety amid the COVID-19 epidemic. These narratives illuminate the intricate interactions between social, psychological, and environmental elements that influence mental health and well-being during times of crisis, via first-hand accounts of suffering, resiliency, and recovery.

#### Coping through creative expression and self-care

During the COVID-19 epidemic, the theme “Coping through Creative Expression and Self-Care” examines the healing potential of creative pursuits and self-care routines in promoting the mental health of older women. This theme explores the various ways that older women deal with the difficulties of loneliness, anxiety, and bereavement by using the therapeutic power of creative expression—such as painting, writing, music, and gardening—as well as self-care practices like mindfulness, meditation, and physical activity. This subject highlights the significant role that artistic endeavours and self-care practices have in developing resilience, self-awareness, and inner strength in the face of hardship via personal accounts and research findings. A 63 years old mentioned:

After the COVID-19 pandemic began, I had a great phase of introspection and self-finding. Surrounded in chaos and uncertainty, I closed myself away at home and prayed for half-openness in my garden. Each morning, I would step outside to look after my plants, the rhythm of nature exerting its own calming influence on my turbulent mind.

The narrative emphasises the therapeutic advantages of artistic pursuits as a way to deal with loneliness and mental suffering, such as painting and gardening. Empirical evidence bolsters the idea that leisure pursuits such as gardening and art therapy help foster calmness, minimize stress, and elevate the emotional state of senior citizens (34). Another participant narrated;

In search of peace and comfort, I delved into creative pursuits. Each morning, I would lace up my trainers and go on long hikes in the open air, letting the rhythm of my footfalls slow down my racing mind. Walking did not only lend me a measure of tranquillity, but in the worst times it kept me going at all. Every afternoon I would take up residence in my art studio, where I was surrounded by the rich textures and colours of my paintings. It wasn't until painting that I could work through all the pain and discover beauty floating amid turmoil, in a kind of language that transcended words.

The significance of self-care activities like walking, drawing, and meditation in fostering resilience and emotional well-being is highlighted by this narrative. Studies reveal that taking walks in the outdoors, meditating, and creating art may improve mood, lessen anxiety, and foster a sense of balance and inner serenity (35). These accounts illustrate the various ways that self-care and artistic expression support older women's emotional fortitude and wellbeing under trying circumstances. Through facilitating emotional release, interpersonal growth, and self-awareness, these coping mechanisms enable senior women to face hardships with dignity, bravery, and fortitude.

#### Finding strength in resilience and social support

This subject looks at how older women deal with challenges, overcome barriers, and find belief in the face of uncertainty by using their inner capacity, adaptability, and support systems. For instance, a 72 years old retiree women mentioned;

When I felt lonely and isolated, I turned to friends and family in my support network. Their words of support and kindness comforted me. Although we did not get to see each other face-to-face, we bridged the divide through phone calls and virtual gatherings. In the most shadowy day, we leaned on each other, a lifeboat of connection and support that bridged the physical world.

The narrative is consistent with secondary research showing that older people use their social networks and resilience to help them deal with the pandemic's problems (36). According to research, maintaining social ties—even virtual ones—can protect older persons from loneliness and enhance their psychological wellbeing (37). The participant narrative emphasises the value of social support and resilience in creating mental health and a feeling of community among older women who are confronting difficulty and uncertainty. A 75 years old widow reflected;

From phone calls to services, we gradually gathered together and became a closely-knit community gathering for something in common at its heart. Confronting adversity, resilience and support offered me hope. I also discovered social support, the existence of which can strengthen us in periods of doubt and difficulty. My support network consisted of friends, neighbors, and community members; simple acts of charity and compassion made for moments of comfort here and there.

This narrative correlates with subsequent studies emphasising the value of social support in mitigating the negative effects of loneliness and grief on the well-being of senior citizens. Studies show that throughout the pandemic, individuals with robust social support networks experienced fewer cases of anxiety and despair and better levels of resilience (38). During the COVID-19 epidemic, the participants of this study highlight the transformational impact of social support and resilience in promoting older women's emotional well-being and sense of community. Through first-hand accounts of bravery, fortitude, and connection, these tales emphasise how crucial it is to lean on one's inner strength and support systems to get through difficult times and maintain optimism in the face of uncertainty.

## Discussion

The current study “Loneliness and Social Isolation: Exploring the Experiences of Older Women during the Pandemic in terms of Social Connection, Feeling of Loneliness, and the Impact on Mental Health and Well-Being” captures the complex difficulties older women encountered during the COVID-19 pandemic. With an emphasis on the experiences of older women in this historic period, the conversation examines the interconnected aspects of social connection, loneliness, and the influence on mental health. The findings are discussed in terms of two main themes with each having its sub-themes. The theme of “Social Connection amidst Physical Distancing Measures” illuminates the resilience and adaptability of individuals in maintaining meaningful relationships despite the challenges posed by the COVID-19 pandemic. From the analysis, it was found that social distancing made it necessary to hold virtual conferences, followed by a surge of every kind of digital interaction. Above all else, older women demonstrated an extraordinary capacity for learning when it came to using technology to stay in touch with family and friends. Video calls, social media, and messaging apps were all lifelines between loved ones separated by great distances. Through virtual exchanges, older women in this study were able to achieve a “feeling” of attachment, creating a kind of smooth “transition” from loneliness and isolation to companionship. Other studies also indicate that technology-mediated communication can improve older individuals' general well-being, lessen their feelings of loneliness, and increase social support (39). To guarantee equal access to virtual communication tools, specialised interventions are necessary, as certain older persons continue to face obstacles related to digital literacy and technological access. A re-evaluation of the value of intimate connections was spurred by the epidemic, with an emphasis on the superiority of quality over number in social interactions. People withdrew from social events and travelled less, spending more time with close friends and family and becoming more introspective. Common activities like cooking together, doing hobbies together, and thinking back on old time's established stronger ties and promoted a more intimate relationship. The chance to re-establish deeper connections with loved ones was especially welcomed by older women, who treasured their time spent together even in the face of hardship. Strong interpersonal ties have been linked to improved mental and physical health outcomes, according to research, underscoring the importance of closeness and social support in times stressful events (40). Re-established gratitude for close connections may persist as people recover from the pandemic, providing a source of strength and happiness in the post-pandemic environment. Social routines had to be disrupted, which meant learning new methods to interact and connect with others. Participants of this study demonstrated inventiveness and adaptability in navigating shifts in social norms and expectations, whether they were part of virtual festivities or outdoor get-togethers. Particularly older persons showed resilience in adapting to evolving social dynamics and embracing connection possibilities while still following safety precautions. The epidemic acted as a spark for rethinking customs and social routines, encouraging creativity and flexibility in interpersonal relationships. However, the interruption of well-known patterns also presented difficulties, resulting in emotions of loss and confusion. Research suggests that having a sense of regularity and structure is crucial for wellbeing, which emphasises the need for measures that help people adjust to changes in social routines (41). People will continue to negotiate the shifting social interaction environment as society settles into a new normal, relying on their resiliency and inventiveness to create meaningful relationships in a world that is always changing (42).

The theme of “Impact on Mental Health and Well-being” delves into the profound emotional effects experienced by individuals, particularly older women, during the COVID-19 pandemic. This discussion will explore three subthemes: “Struggles with Anxiety and Depression,” “Coping through Creative Expression and Self-Care,” and “Finding Strength in Resilience and Social Support.” From the participant's perspective of this study, the COVID-19 pandemic has made mental health issues worse and caused worry and despair in some people, especially in older women. Psychological suffering has increased as a result of the pandemic's uncertainty, fear, and isolation. Particularly at crisis are older women, who may already be susceptible because of things like social isolation, long-term medical issues, and caring obligations. Other studies have also found that the pandemic has contributed to a notable rise in the prevalence of anxiety and depression among older persons, with symptoms being exacerbated by feelings of helplessness and loneliness (43). To address these issues and promote older women's well-being, access to mental health interventions and support has become essential. However, in this study the participants manifested coping mechanisms to deal with mental health and well-being. The participants of this study have resorted to self-care and artistic expression as coping techniques to support mental well-being and reduce stress in the face of the pandemic's challenges. Taking part in creative endeavours including writing, music, art therapy, gardening, and mindfulness meditation has given people a way to process their feelings, develop self-awareness, and build resilience. These findings have also been found in other studies (44). Through these techniques, people can develop a feeling of agency in overcoming hardship and take charge of their mental health. Studies indicate that engaging in self-care practices is essential for reducing the negative effects of stress and fostering psychological fortitude in older adults (45). Older women can improve their capacity to manage the pandemic's challenges and preserve their emotional health by making self-care a priority (46). It's also noteworthy that researchers in this study found that social support and resilience have been identified as crucial defence mechanisms against the pandemic's detrimental effects on mental health and general wellbeing. Particularly older women have shown incredible resiliency in adjusting to the difficulties brought forth by the epidemic and drawing support from their social networks. According to research, social support is essential for maintaining psychological health and preventing feelings of isolation and loneliness. Older women can count on their social networks for companionship, practical help, and emotional support by cultivating relationships with friends, family, and neighbours. Furthermore, developing resilience via adaptive coping techniques like acceptance, positive reframing, and problem-solving might enable older women to face the pandemic's uncertainty with bravery and resilience.

To better understand the pandemic's impact on older women, future studies should give precedence to longitudinal designs that can follow changes over time and investigate how different aspects of identity—like race or economic status—interact with one another. Moreover, scientists ought to examine which technologies are adopted under what circumstances; which factors promote resilience in people who don't have much support around them; and what kinds of interventions work best when it comes to improving wellbeing among marginalized populations. Finally, policymakers should evaluate programs intended for them as well as community-based systems aimed at supporting this vulnerable group. Such an analysis would help meet the unique needs of older females while also informing future action.

### Limitations of the study

There are a few important limitations to be aware of, even if our study shed light on the experiences of older women during the COVID-19 epidemic. First and foremost, it's probable that participants' answers were shaped by their subjective perceptions of their experiences or social desirability. Furthermore, because research participants who volunteered and those who did not would have varied consistently, the recruiting method might have resulted in selection bias. Moreover, the absence of longitudinal data restricts our comprehension of the dynamic character of these phenomena by making it difficult to follow changes in mental health outcomes, social connectedness, and loneliness over time. Furthermore, participants’ experiences may have been impacted by contextual factors including socioeconomic position, geography, and cultural differences, which might have an impact on how broadly our findings can be applied to different groups.

## Conclusion

In the pandemic, older people were living in enforced social isolation. Research on older women has made it clear that they suffer from multiple problems when they try to maintain social relationships. Finding inner peace is necessary to manage their social isolation. Many difficulties are posed by the infinite number of challenges faced by older women who feel lonely, which include keeping in touch with others and treating mental health issues. The coronavirus situation has heightened pre-existing vulnerabilities and enhanced the negative effects of social isolation on older women, forcing targeted interventions and support systems, to meet their different needs. Through this study, we now know that social networks play a pivotal role in helping mitigate feelings of loneliness and supporting the mental health of older women. Embracing technology for virtual interactions, rediscovering the value of intimacy, and adjusting to changes in social rhythms have become important coping mechanisms for building social networks and resilience against adversity. Nevertheless, many older women still feel lonely, and the resultant mental torments remain a painful reality. The solution to loneliness is for everyone else to work on it together. It means intervention in an enormous variety of forms-social, psychological, and environmental. Including community-based programs, mental health support services, and even peer support groups. To this end, what we most urgently need is take good care of the older women, create inclusive and supportive environments, promote opportunities for social engagement and connection, and increase access to mental health services. Older women have experienced the pandemic in ways distinct from others.

## Data Availability

The raw data supporting the conclusions of this article will be made available by the authors, without undue reservation.
